# The Effects of UV Light on the Chemical and Mechanical Properties of a Transparent Epoxy-Diamine System in the Presence of an Organic UV Absorber

**DOI:** 10.3390/ma10020180

**Published:** 2017-02-14

**Authors:** Saeid Nikafshar, Omid Zabihi, Mojtaba Ahmadi, Abdolreza Mirmohseni, Mojtaba Taseidifar, Minoo Naebe

**Affiliations:** 1 Department of Applied Chemistry, Faculty of Chemistry, University of Tabriz, Tabriz 51666, Iran; s_nikafshar@yahoo.com (S.N.); a.mirmohseni@hotmail.com (A.M.); 2 Institute for Frontier Materials, Deakin University, Geelong, Burwood, VIC 3125, Australia; 3 Department of Chemical Engineering, Isfahan University of Technology, Isfahan 84156/83111, Iran; mojtaba.ahmadi@ce.iut.ac.ir; 4 School of Physical, Environmental & Mathematical Sciences, UNSW, Canberra, ACT 2610, Australia; m_taseidifar@yahoo.com

**Keywords:** epoxy resin, UV absorber, photodegradation process, Tinuvin 1130

## Abstract

Despite several excellent properties including low shrinkage, good chemical resistance, curable at low temperatures and the absence of byproducts or volatiles, epoxy resins are susceptible to ultra violet (UV) damage and their durability is reduced substantially when exposed to outdoor environments. To overcome this drawback, UV absorbers have been usually used to decrease the rate of UV degradation. In this present study, the effects of UV light on the chemical, mechanical and physical properties of cured epoxy structure, as well as the effect of an organic UV absorber, Tinuvin 1130, on the epoxy properties were investigated. Chemical changes in a cured epoxy system as a result of the presence and absence of Tinuvin 1130 were determined using Fourier transform infrared spectroscopy (FT-IR) analyses. The effect of Tinuvin 1130 on the surface morphology of the epoxy systems was also investigated by scanning electron microscopy (SEM) imaging. Additionally, the glass transition temperatures (*T*_g_) before and during UV radiation were measured. After an 800 h UV radiation, mechanical test results revealed that the lack of the UV absorber can lead to a ~30% reduction in tensile strength. However, in the presence of Tinuvin 1130, the tensile strength was reduced only by ~11%. It was hypothesized that the use of Tinuvin 1130, as an organic UV absorber in the epoxy-amine system, could decrease the undesirable effects, arising from exposure to UV light.

## 1. Introduction

Nowadays, various polymeric materials are commercially available and used in many applications such as protective coatings, civil engineering materials, aerospace composites, automotive fields, and marine industries [1,2,3,4]. The durability of materials is a key factor for many applications which affects the performance of polymer-based materials. The sun is the major source of energy for the earth and emits a vast range of wavelengths in which only ultraviolet (UV) light, visible and infrared light (IR) reach the earth [5]. Ozone layer filters emit UV wavelengths from 100 to 315 nm. Definitely, the ozone layer completely eliminates UVC (100–280 nm) and most of UVB (280–315 nm) and only UVA (315–400 nm) and a small amount of UVB reach the earth [6]. The UV radiations are harmful to polymers, a phenomenon known as photodegradation is initiated which affects the physical, mechanical and chemical properties of polymers [7,8,9,10,11]. UV light of sunlight forms free radicals on the surface of polymer-based materials [12]. These radicals are extremely active in attacking the polymeric structures. The energy of UV light is usually higher than the chemical bonds strength e.g., C–C, O–O, H–O, and C–N in polymers. Therefore, these chemical bonds are prone to break and photodegradation occurs [13]. Other environmental factors including humidity, oxygen, temperature, and pollutants can intensify the rate of photodegradation significantly [14,15,16].

**Figure 1:**
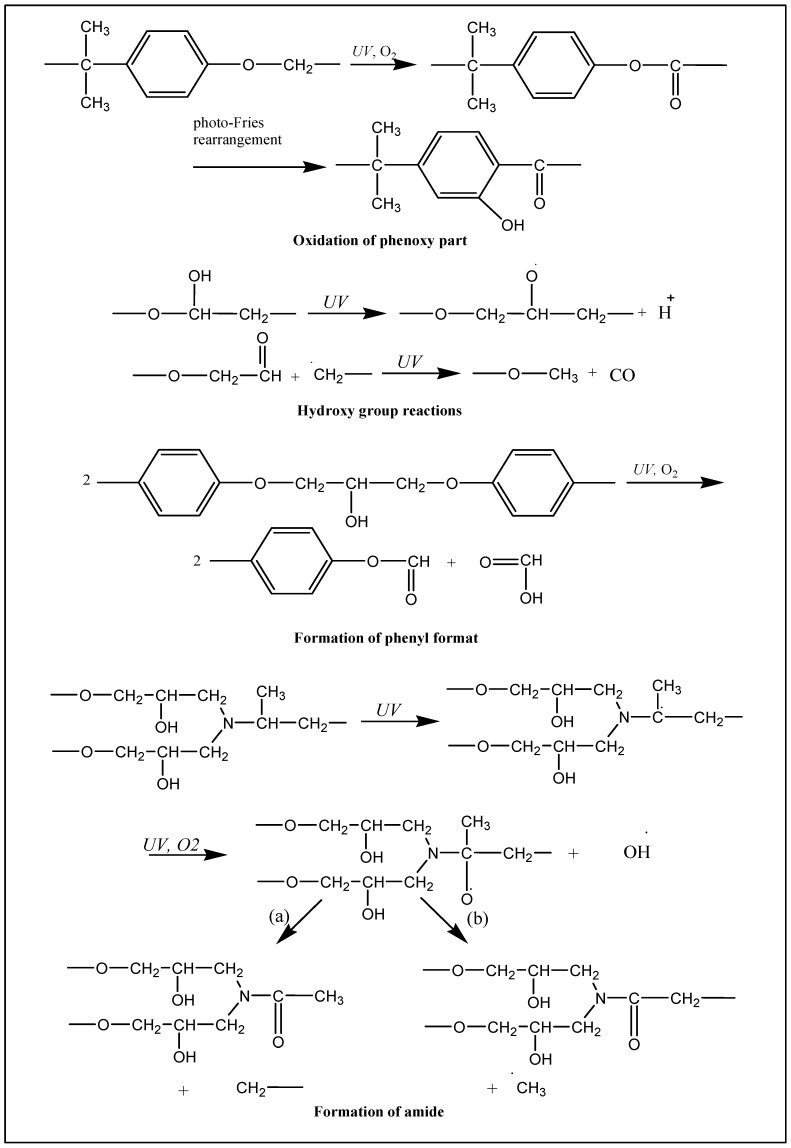
Various mechanisms of UV degradation in the epoxy system.

To avoid or reduce photodegradation, inorganic and organic UV absorbers, as well as stabilizers have been used [17,18,19]. The mechanism of these UV absorbers against UV light varies from one material to another. Inorganic UV absorbers often scatter light, while organic UV absorbers convert UV light to heat, higher wavelengths, and radical interceptors (HALS), capturing free radicals [16,19,20]. Compared with inorganic UV absorbers, organic UV absorbers have higher efficiency but they have less durability due to their evaporation and migration to the surface of polymers [21,22]. In addition, inorganic UV absorbers such as ZnO and TiO_2_ have broad absorptions in the UV range and even in the visible range, whereas organic UV absorbers only absorb specific wavelengths in the UV range and almost do not have any absorptions in the visible range. It is clear that organic UV absorbers are in high demand and suitable for clear and transparent coatings and polymers [23,24]. Moreover, polymeric coatings with organic UV absorbers, compared with inorganic UV absorbers, are more flexible and have a lower glass transition temperature (*T*_g_). Although epoxy systems have great chemical and mechanical properties, since they are applied in many applications even in the outdoor environment, durability and performance of epoxy structure is reduced dramatically. Herein, yellowing, cracking, and reduction of mechanical properties are inevitable aspects [25]. Epoxy resins possess aromatic groups with a strong absorption in the UV range (about 300 nm). This makes epoxy structures vulnerable to UV degradation [26]. When oxygen molecules in the air are exposed to UV light radiation, oxygen radicals are produced. Radicals are also formed from broken bonds, which are highly reactive and have a very short life [27]. These formed radicals attack the surface of the epoxy coatings and react with them. There are various mechanisms proposed for UV degradation of cured diglycidyl ether bisphenol A (DGEBA), as presented in Figure 1.

**Figure 2:**
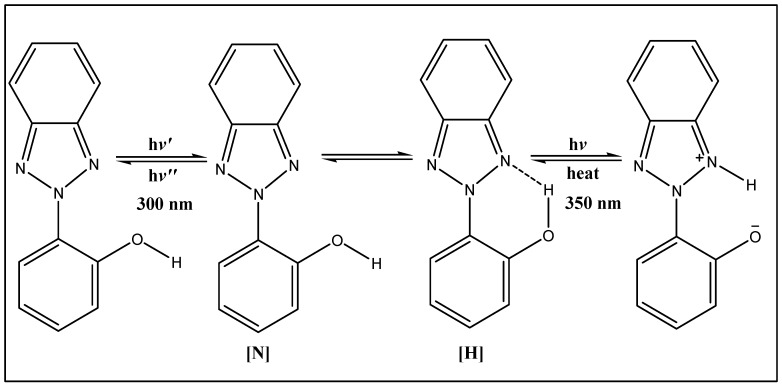
The reaction between a benzotriazole compound and UV light.

Currently, several organic UV stabilizers with different maximum absorption bonds are produced commercially. According to the chemical structures of organic UV stabilizers, they can be divided into three categories: benzotriazoles, benzophenones, and hindered amine light stabilizer (HALS). They have different reactions with UV light. When benzotriazoles are exposed to UV light, they, as organic UV absorbers with aromatic parts, can behave in the three following ways: changing the conformational structure, emitting higher wavelength, and converting UV light to heat [24]. The two conformations of a benzotriazole compound, (2(2-hydroxyphenyl)2*H*-benzotriazole), with UV light are shown in Figure 2. The [H] conformation of the molecule, which directly inclines itself to the hydrogen bonding, has the ortho-hydroxyl group aligned to the cisoid arrangement, while the ortho-hydroxyl group is aligned in a transoid arrangement in another isomer [N]. In the [H] isomer, UV light (350 nm) is converted to heat, while in the [N] isomer, UV light is emitted as a longer wavelength [20]. When a benzophenone compound is exposed to UV light, it is excited to the single state and the proton of the hydroxyl group is transferred to the carbonyl group. The efficiency of benzophenones for absorbing UV light is lower than that of benzotriazoles [11]. On the other hand, HALSs do not absorb UV light yet they act as polymer degradation inhibitors. They either stop or at least slow down the degradation reaction rate by scavenging produced radicals [28]. Organic stabilizers are produced under the commercial name of Tinuvin. For instance, Tinuvin 5151, Tinuvin 326, Tinuvin 328, and Tinuvin 1130 are based on benzotriazoles with different substituents, while Chimassorb 81 is based on benzophenone. It is worth mentioning that Chimassorb 944 and Tinuvin 770 are classified as HALSs. 

Actually, through modification of the chemical structure of UV absorbers, various UV absorbers with different maximum bonds can be synthesized [20,29]. UV absorbers can be distinguished by absorption bonds, the phase of materials, melting points, multiple colors, and miscibility. Therefore, based on polymers types and their structures, several types of UV absorbers can be used. In recent studies, UV performance of different organic UV absorbers in polymers has been investigated. Tinuvin 1130 and Tinuvin 5151 were used as UV absorbers in clear water-borne coatings (acrylic-polyurethane blends) and it was found that Tinuvin 1130 was more effective than Tinuvin 5151 in terms of protection against UV light [19]. Tinuvin 1577 was added into polyethylene terephthalate (PET) and it was found that addition of Tinuvin 1577 effectively reduced the photodegradation of PET [30]. Jia et al. [31] reported high UV stability of polypropylene with the addition of Tinuvin 328. The effect of Tinuvin 384 on the isocyanate type of acrylated urethane oligomer was investigated by Lee and Kim [32] and they observed an improvement in photodegradation. Liu et al. [33] investigated the effect of four types of UV stabilizers e.g., Tinuvin 326, Chimassorb 81, Tinuvin 770, and Chimassorb 944 on ethylene-octene copolymer. They found that the UV performance of Tinuvin 770 and Chimassorb 944 was higher than that of Chimassorb 81 and the UV performance of Tinuvin 326 was the lowest. 

In this study, the photodegradation process of epoxy in the presence and absence of Tinuvin 1130 was investigated and the chemical as well as mechanical properties were studied. Since epoxy resins have a high amount of aromatic rings in their structures which can absorb UV light around 300 nm, Tinuvin 1130 was selected as a UV absorber working at UV light in the ranges of 300–350 nm and 350–390 nm optimally. It was believed that this UV absorber could protect polymeric matrices against UVA and UVB ranges. In addition, Tinuvin 1130 which is a viscous liquid can be applied as a UV absorber for epoxy resin without using any solvents which may negatively affect the final mechanical properties of the epoxy. Moreover, Tinuvin 1130 almost does not have any absorption in the visible area; therefore, it can be used for transparent polymeric structures. To the best of our knowledge, there have been no reports investigating chemical, physical, and mechanical properties of epoxy with and without Tinuvin 1130 as UV absorber in the literature. 

## 2. Experimental

### 2.1. Materials

**Figure 3:**
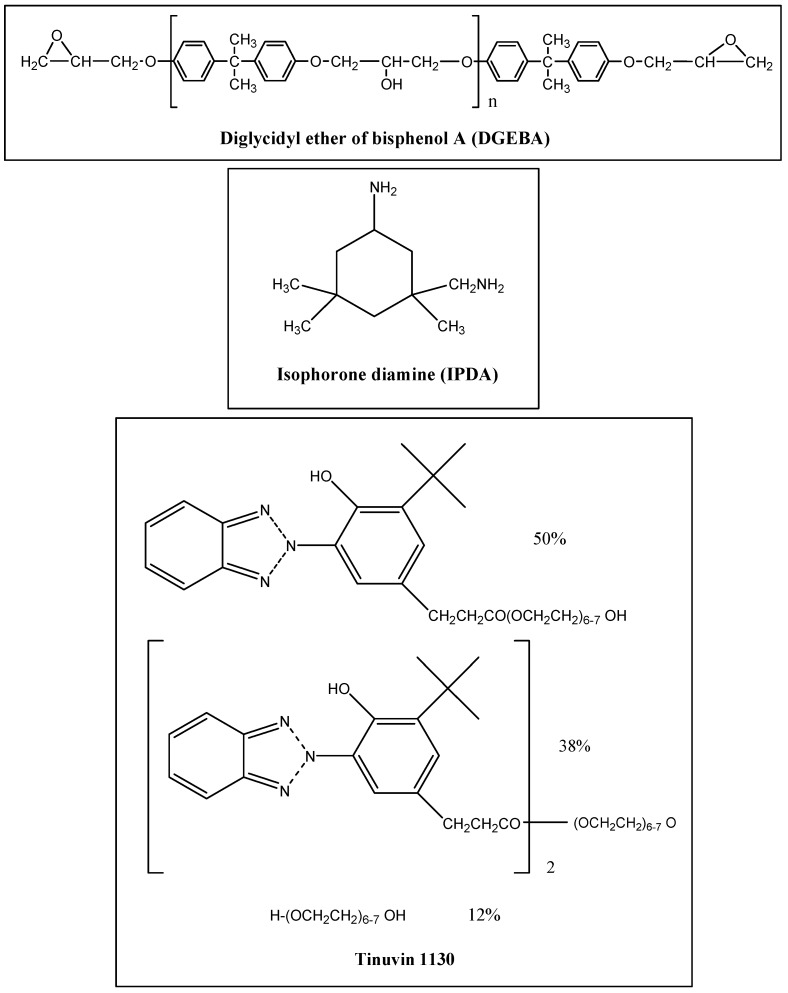
Chemical structures of diglycidyl ether of bisphenol A (DGEBA), isophorone diamine (IPDA) and Tinuvin 1130.

Liquid diglycidyl ether of bisphenol A (DGEBA, Epon 828, Hexion, Columbus, OH, USA) with an epoxy equivalent weight of 185–192 g/eq was supplied from E.V Roberts. Epikure F205 was purchased from Hexion used as a curing agent. This curing agent was based on isophorone diamine (IPDA) which was further modified to cure the epoxy resin at room temperature. Tinuvin 1130 was obtained from Ciba Specialty Chemicals (Basel, Switzerland). The chemical structures of DGEBA, IPDA, and Tinuvin 1130 are shown in Figure 3.

### 2.2. Methods

The average epoxy molar mass (EMM) of Epon 828 according to the manufacturer was 188 g/eq. The amine hydrogen molar mass (AHMM) of Epikure F205, which was employed as a curing agent, was reported to be 105 g/eq. Therefore, each amine hydrogen reacts with one epoxy group and the stoichiometric ratio of the curing agent to epoxy resin for curing reaction can be determined according to AHMM/EMM = 0.56. To prepare samples, 10 g of epoxy resin was stirred with 5.6 g of Epikure F205. All samples were prepared according to the obtained ratio of 1:0.56 epoxy resin and F205, respectively, and only the quantity of Tinuvin 1130 in the samples was altered. Tinuvin 1130 was yellow to light, an amber, viscous liquid, and the proposed amount of Tinuvin 1130, according to its manufacturer, was 3% for an epoxy resin. This amount of Tinuvin 1130 was high because the UV degradation process can be accelerated with decreasing thickness of the coating, and consequently, it needed to be used in a higher quantity [34]. For FTIR spectroscopy and UV-visible spectroscopy analyses, DGEBA, Epikure F205, and 3% Tinuvin 1130 (only for sample B) were mixed for 3 min, coated onto a polyethylene sheet and after 8 h at 25 °C, the formed coating was separated from the polyethylene sheet. The thickness of the coating was ~0.5 mm, and the coating was cut into small pieces of about 2 × 2 cm and stored in a dark place for 7 days at 25 °C. This was mainly due to the fact that some samples had UV absorbers (sample B) which were sensitive to light. Samples were labeled as A_1_, A_2_, A_3_, A_4_ and B_1_, B_2_, B_3_, and B_4_ and placed under UV light. Subscribes 1, 2, 3, and 4 meant that samples were exposed to UV light for 0, 250, 500, and 800 h, respectively. To measure the glass transition temperature (*T*_g_), 5 mg of each prepared film samples for Fourier transform infrared (FT-IR) spectroscopy analysis, before and after UV radiation (various exposed time 250, 500, and 800 h), was put into an aluminum pan. Samples for tensile test and Izod impact were prepared as follows: DGEBA, Epikure F205 and 3% Tinuvin 1130 (only for sample B) were stirred for 3 min and the mixture was then poured into a silicon mold. To complete the curing reaction, the samples were cured for 8 h at room temperature, and then specimens were removed from the mold and kept in a dark place at room temperature for 7 days. Epoxy resin, Epikure F205, and 3% Tinuvin 1130 (only for sample B) were mixed for 3 min and then the mixture was poured onto eight aluminum plates, following that the pull-off strength was measured for each prepared coated sample on the aluminum plates. Each sample was tested four times and the average was reported. The surfaces of the prepared epoxy films were also studied by scanning electron microscopy (SEM, MIRA3 FEG-SEM, Tescan, Brno, Czech Republic). Hitachi F40T10/BL UVB lamp 40 W with a length of 120 mm was used for radiation (Hitachi, Tokyo, Japan). The lamp, installed in a fully closed aluminum box, started to emit wavelength ranging from UV to visible, but the main emitted wavelength was around 290–320 nm (UVB). Samples were placed under the UV light radiation with 5 cm distance from the lamp. To regulate the temperature, a fan was placed on one side of the aluminum box and the temperature was kept at about 25 °C during UV radiation. The photodegradation process continued for 800 h. Before radiation and after certain time periods e.g., 250, 500, and 800 h various tests were performed on the samples. 

### 2.3. Analysis Techniques

FT-IR analyses were conducted using a Tensor 27 (Bruker, Billerica, MA, USA) with 40 scan average at a resolution of 4 cm^−1^. In this study, FT-IR spectroscopy was used to study the functional groups and chemical structures of samples before and after UV radiation. To measure the absorption in UV and visible ranges (200–400 and 400–700 nm), a UV-visible spectrophotometer was utilized (Shimadzu UV-1700 pharmaspec, Shimadzu, Kyoto, Japan). Differential scanning calorimetry (DSC) analyses (Linseis PT10, Linseis, Robbinsville, NJ, USA) were used to determine *T*_g_. For each sample, a non-isothermal test at a heating rate of 10 °C/min was carried out at a temperature ranging from 0 to 150 °C under a nitrogen flow of 40 mL/min. A tensile strength test was conducted according to ASTM D638 with a Shimadzu 20 kN-testing machine. Specimen dimensions were selected according to type 1 of ASTM (165 × 19 × 3.2 mm^3^). The crosshead speed was 2 mm/min. At least five samples for each condition were tested. A Posi test AT-M from Deflesco was used to measure pull-off strength according to ASTM D4541. Measurements were performed after 0, 250, 500, and 800 h UV radiation. At least five measurements were conducted. To evaluate the effect of UV light on the Izod impact strength of samples, five samples from each specimen were prepared. The dimensions of samples were 63.5 × 12.7 × 7.2 mm^3^, according to ASTM D256 using a Zwick/Roell 6103 impact tester (Zwick/Roell, Ulm, Germany). The test was carried out after 0, 250, 500, and 800 h UV radiation. This test was repeated five times for each sample. To study the effect of UV light on the morphology of the epoxy samples, epoxy coatings were examined using SEM (MIRA3 FEG-SEM, Tescan). Samples were gold coated and an accelerating voltage of 5.00 KV was employed. 

## 3. Results and Discussion

**Figure 4:**
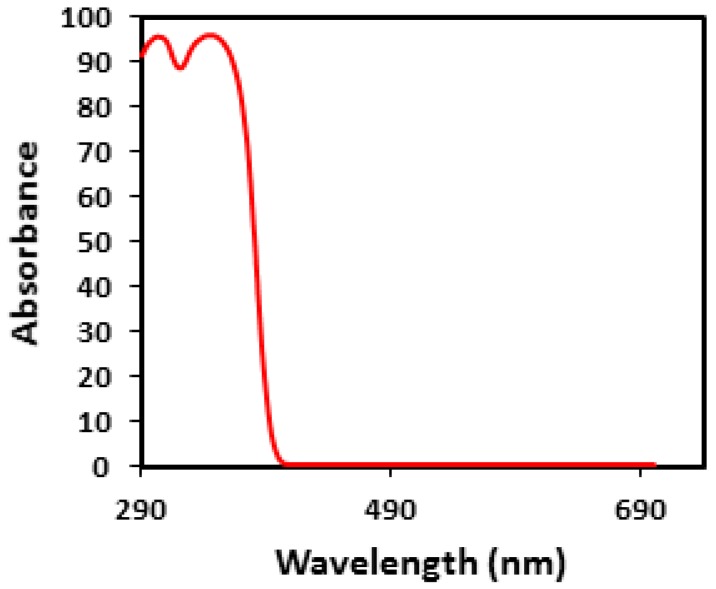
The UV-visible spectrum of Tinuvin 1130.

As presented in Figure 4, Tinuvin 1130 had two absorption peaks at 305 and 345 nm and was used as an organic UV absorber for this work. Because Tinuvin 1130 does not have any absorption in the visible area (400–700 nm), it can be used in transparent polymers like epoxy. Based on further investigation, it was determined that Tinuvin 1130 as an organic UV absorber played a major role in protecting the epoxy structure against UV light. Experimentally, it is proposed that Tinuvin 1130 can not only preserve the chemical properties but can also prevent or at least reduce the drop in the mechanical properties. 

### 3.1. FT-IR Analysis

**Figure 5:**
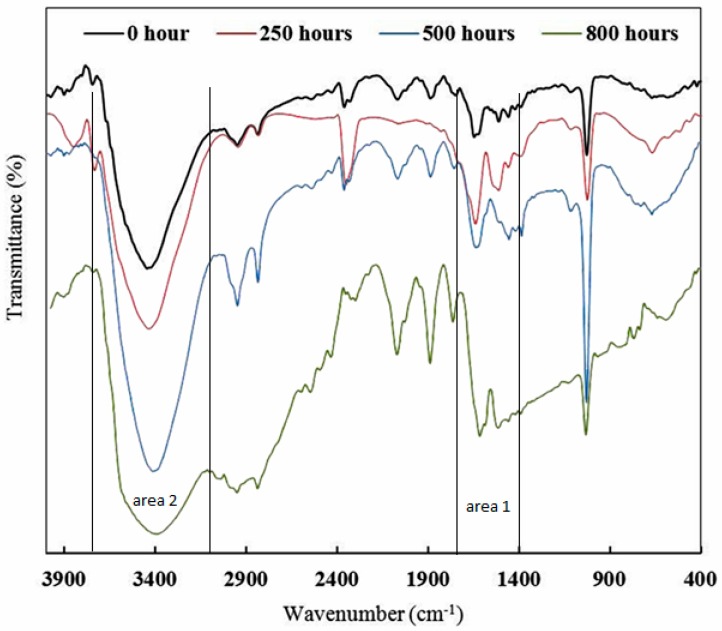
FT-IR spectra of sample A (epoxy resin and Epikure F205) after 0, 250, 500, and 800 h of UV radiation.

**Figure 6:**
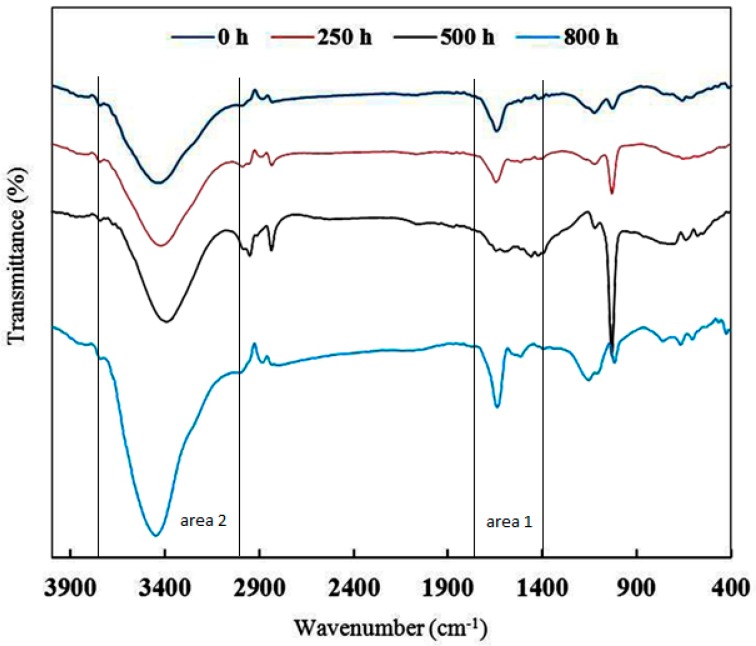
FT-IR spectra of sample B (epoxy resin and Epikure F205 with 3% Tinuvin 1130) after 0, 250, 500, and 800 h of UV radiation.

FT-IR spectroscopy is a common method for evaluating chemical changes during the UV degradation process in epoxy and polymeric systems [18,35,36,37,38]. The effects of UV light on the epoxy structure can be studied by FT-IR. Figure 5 shows FT-IR spectra of sample A (epoxy resin, Epikure F205) before and after 250, 500, and 800 h of UV radiation. To study the photodegradation phenomenon, two areas of FT-IR spectra should carefully be considered. The areas of 1600–1800 cm^−1^ (area 1) and 3200–3700 cm^−1^ (area 2) are related to carbonyl groups and hydroxyl groups, respectively [39,40]. Before UV irritation (0 h), the absorption intensity in areas 1 and 2 (carbonyl and hydroxyl groups) was low, while after 250-h UV irradiation, the absorption intensity in areas 1 and 2 increased. After increasing the UV irradiation time to 500 h, the level of absorption bands in area 1 and area 2 still increased. According to the proposed mechanisms in Figure 3, more carbonyl and hydroxyl groups are generated during UV degradation. It is clear that after 250 and 500 h of UV radiation, absorption in the mentioned areas (areas 1 and 2) were enhanced, indicating the photodegradation process. After increasing the UV radiation time to 800 h, not only absorption bands in areas 1 and 2 increased but also an absorption band in the range 2400–3400 cm^−1^ appeared which was attributed to carboxylic acid groups [40]. It can be said that after 800 h UV radiation, more carboxylic acid groups were formed, which can be considered as an advanced UV degradation step. As seen in Figure 6, after 250 and 500 h of UV radiation, no significant changes were observed in the absorption intensity of carbonyl and hydroxyl areas in sample B which contains 3% Tinuvin 1130. In this sample, after 800 h UV radiation, absorption intensities in the 1600–1800 and 3200–3700 cm^−1^ areas (carbonyl and hydroxyl groups) increased slightly. This level of degradation was a preliminary step in which sample B still did not reach the advanced UV degradation step. Sample B was protected against UV degradation up to 800 h of radiation as a result of Tinuvin 1130. The absorption intensities of carbonyl and hydroxyl functional groups (areas 1 and 2) in Figure 5 and Figure 6 further confirmed the role of Tinuvin 1130 as a UV absorber.

**Figure 7:**
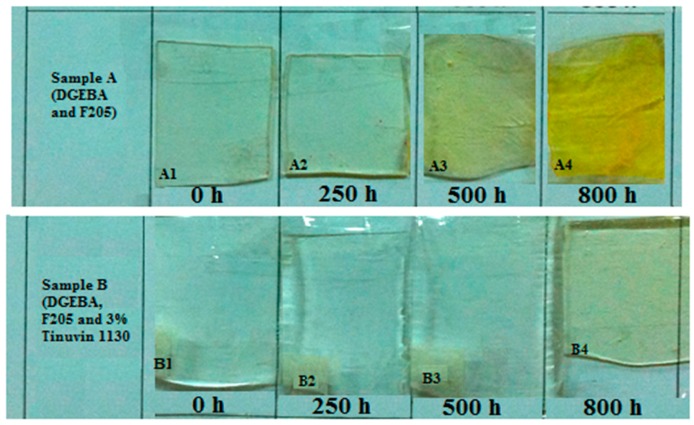
Optical images of samples A and B after 0, 250, 500, and 800 h of UV radiation.

The optical images of samples A and B after 0, 250, 500, and 800 h UV radiation are shown in Figure 7. In sample A, yellowing after 500 h UV radiation was clearly seen and the yellowing intensity reached the highest level after 800 h of UV radiation. While only after 800 h of UV, a low intensity of yellowing was seen in sample B, indicating an effective protection of sample B against UV light due to the organic UV absorber (Tinuvin 1130).

### 3.2. UV-Vis Analysis

**Figure 8:**
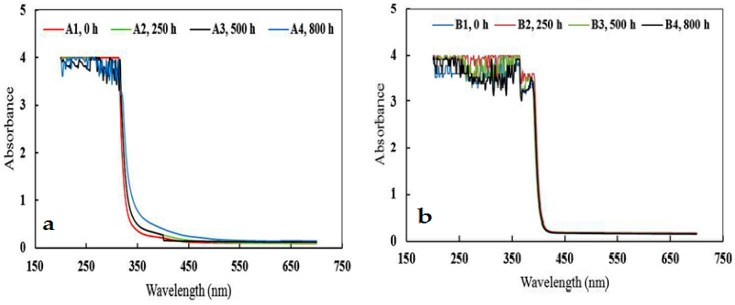
(**a**) UV-Vis spectra of sample A (epoxy resin and Epikure F205) after 0, 250, 500, and 800 h of UV radiation; (**b**) UV-Vis spectra of sample B (epoxy resin and Epikure F205 with 3% Tinuvin 1130) after 0, 250, 500, and 800 h of UV radiation.

UV-Visible spectroscopy has been used to study the UV degradation process, particularly for transparent polymers and coatings [19,39,41]. Due to the presence of several aromatic groups in the epoxy structure, the absorbance intensity in the UV range (200–320 nm) was high. As can be seen in Figure 8a, when the epoxy coating was exposed to UV light radiation, the UV intensity was very high at first. However, after 250 and 500 h of UV radiation, the absorbance level was reduced because the aromatic groups were derogated or had undergone rearrangements. After 800 h, the absorbance intensity, in comparison to 250 and 500 h, was increased due to the formation of carboxylic acids. Certainly, this was an advanced UV degradation step, forming phenyl formate carboxylic acid groups in which the phenyl groups were in resonance and consequently caused intensification of the reduced absorbance. According to Figure 8b, by adding Tinuvin 1130 as a UV absorber, the domain of the UV absorbance was extended from 200 to 400 nm which was almost complete absorbance in the UV range. Absorption behaviors of sample B in the UV area were comparatively contrary to the sample A. After UV radiation for 250 and 500 h, the absorbance intensity increased, and after 800 h of UV radiation, the absorbance intensity decreased. 

A spectro-colorimeter is usually used for evaluating color changes in polymers [42,43]. In this work, in order to evaluate color changes in the samples under UV radiation, the area under the visible range (400–700 nm) absorption spectrum was used. In fact, using this method, color changes can be interpreted as quantitative data. These results obtained from analyzing data by OriginPro 8.6 software are presented in Table 1. As can be seen, the visible area (400–700 nm) increased with increasing UV radiation time in the sample A, indicating color changes in the sample. Moreover, the color change from sample of A_3_ to A_4_ was significantly high and severe yellowing occurred. On the other hand, the area of sample B (with Tinuvin 1130) even after 800 h of UV radiation did not change drastically. Tinuvin 1130 not only caused slow UV degradation but also prevented the advanced UV degradation step from occurring. The area of sample B_1_ (before UV radiation) in the visible range was larger than that of A_1_ because Tinuvin 1130 is a yellow to light, amber viscous liquid and when it was added to the mixture of the epoxy resin and Epikure F205, the absorbance intensity in the visible range of the sample B, before UV radiation, was higher than that of the sample A (without Tinuvin 1130). 

### 3.3. T_g_ Evaluation

**Figure 9:**
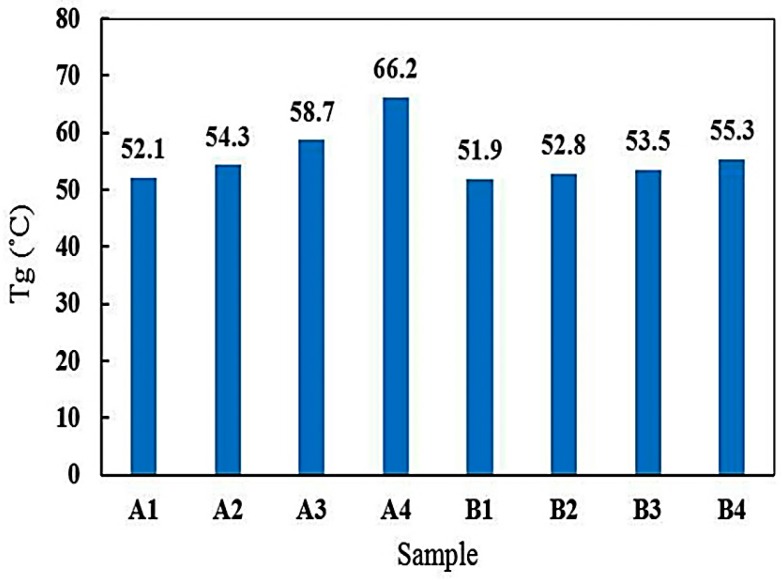
*T*_g_ of samples A and B as a function of UV irradiation time (A: epoxy resin and Epikure F205, B: epoxy resin and Epikure F205 with 3% Tinuvin 1130), (A1: 0 h, A2: 250 h, A3: 500 h, A4: 800 h, B1: 0 h, B2: 250 h, B3: 500 h, B4: 800 h UV irradiation time).

The *T*_g_ was defined as the temperature at which the mechanical properties of polymer changed radically due to the internal movements of the polymer chains [44]. *T*_g_ is one of the most important properties in the evaluation of performance of polymers, which depends on several parameters e.g., molecular weight, branching degree, crosslink degree, bulking substituents, curing temperature, and the polarity of constituents [45,46,47]. Figure 9 shows *T*_g_ values for samples A and B after 0, 250, 500, and 800 h of UV irradiation. It was observed that in sample A (free from UV absorber), with increasing UV irradiation time, *T*_g_ increased, and after 800 h of UV irradiation (A_4_), it reached its maximum value. While in sample B, which contains Tinuvin 1130, the increase of *T*_g_ values continued but to a lesser extent. The increment in *T*_g_ during the UV degradation process made the epoxy coatings brittle. Therefore, after 800 h of UV irradiation, sample B had higher flexibility as a result of a lower UV degradation process, compared to sample A [19]. Increase in *T*_g_ due to the UV radiation is related to formation of polar groups which can hinder chain movements and act as an internal plasticizer [48]. Increasing *T*_g_ reduced the mobility of the polymeric chains, demonstrating the significant contribution of Tinuvin 1130 to the protection of epoxy coating against UV light. 

### 3.4. Tensile Strength

The tensile test was conducted to evaluate the effect of UV light on the mechanical properties of the epoxy polymer [49]. It was hypothesized that polymeric chains and branches were destroyed by UV radiation, affecting the mechanical performance of the epoxy polymers. As shown in Table 2, increasing UV irradiation time decreased the tensile strength and modulus of sample A (epoxy resin and Epikure F204). Nonetheless, it seemed that the tensile modulus was not affected significantly by UV light. After 800 h of UV radiation, tensile strength and elongation at break reduced dramatically. For example, after 800 h, tensile strength and elongation at break of sample A were decreased by ~30% and ~35%, respectively. As reported in Table 2, decreases in tensile strength and elongation at break for sample A after 500 h of UV radiation were accelerated. This could be due to the advanced UV degradation step occurring in this sample. The incorporation of Tinuvin 1130 (sample B) led to a significant slow decrease in tensile strength and elongation at break. After 800 h of UV exposure, the tensile strength and the elongation at break were decreased only by 11.94% and 8.62%, respectively. Therefore, Tinuvin 1130 slowed down the UV degradation rate considerably. 

Chaochanchaikul and Sombatsompop [50] studied the effect of Tinuvin P on mechanical properties of PVC (polyvinyl chloride) with UV irradiation. It was reported that after 720 h of UV irradiation, elongation at break was decreased by 21.01% which was more than twice higher than that reported in this study. In another study, Tinuvin 622 was added into vulcanized ethylene propylene diene monomer rubber (EPDM) and after 672 h of UV irradiation, the tensile strength and elongation at break were reduced by 15.38% and 67%, respectively [51]. 

### 3.5. Izod Impact Strength

**Figure 10:**
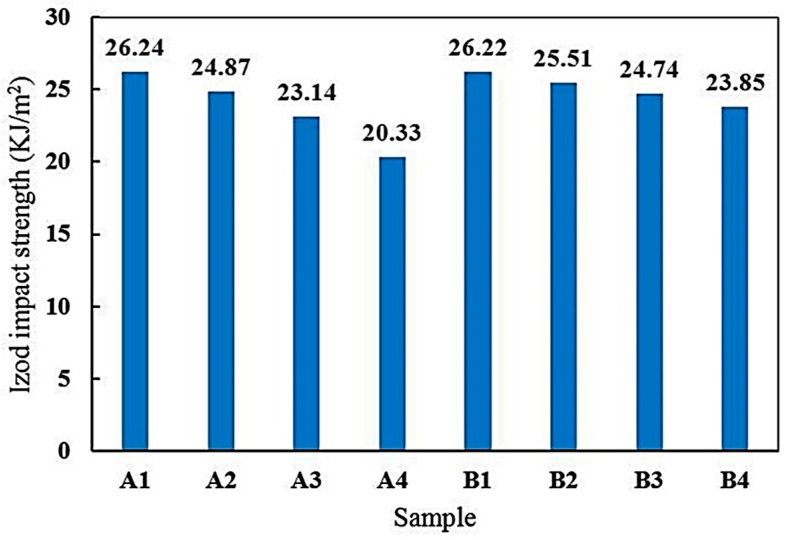
Izod impact strength of samples A (epoxy and Epikure F 205) and B (epoxy resin and Epikure F205 with 3% Tinuvin 1130) after 0, 250, 500, and 800 h of UV irradiation.

Figure 10 shows the impact of Izod strength of samples A (epoxy resin and Epikure F205) and B (epoxy resin and Epikure F205 with 3% Tinuvin 1130) versus UV radiation time. In sample A, UV irradiation reduced Izod impact strength. After 800 h of exposure, Izod impact strength of the samples A and B were reduced by 22.52% and 9.04%, respectively. UV light caused cracks and tore up polymeric chains, resulting in the reduction of Izod impact strength. Nevertheless, UV irradiation damaged sample B less, confirming the positive role of Tinuvin 1130 as an organic UV absorber in epoxy resin. Attwood et al. [52] investigated the effect of UV light on the Izod impact strength of polyolefin blends before and after 800 h of UV irradiation. It was reported that Izod impact strength was reduced roughly by 25% as a result of UV exposure. Other studies have also shown that UV light decreased noticeably the Izod impact strength of various polymeric systems [52,53]. According to the results presented in Figure 10, after 800 h of UV irradiation in the presence of Tinuvin 1130, Izod impact strength was decreased only by 9.1%. 

### 3.6. Pull-Off Strength

Hydroxyl groups in epoxy resin are responsible for adhesion [54]. Therefore, any changes in the concentration of hydroxyl groups in the epoxy structure cause change in pull-off strength. As presented in Figure 1, during UV degradation, some of the primary hydroxyl groups entered into reaction and were converted to other functional groups [9,15]. According to Table 3, increasing UV irradiation time diminished pull-off strength of sample A. It was postulated that, during UV degradation, hydroxyl groups, as major adhesive agents, were either destroyed or involved in side reactions. It was evident that when the concentration of hydroxyl groups was reduced, the pull-off strength decreased. It is worth mentioning that after 800 h of UV radiation (A_4_), pull-off strength decreased significantly, arising from the progress of UV degradation to the advanced stage. On the other hand, changes in pull-off strength for sample B were not as extreme as for sample A, again indicating the protective influence of Tinuvin 1130 over UV damage. Kielmann and Mai [55] examined the effect of UV light on thermosetting N-methylol melamine (NMM) and phenol-formaldehyde (PF) with and without UV absorbers. Pull-off tests on samples revealed that UV absorbers had a positive effect regarding adhesion of epoxy matrix to the aluminum surface. 

### 3.7. SEM Morphology

**Figure 11:**
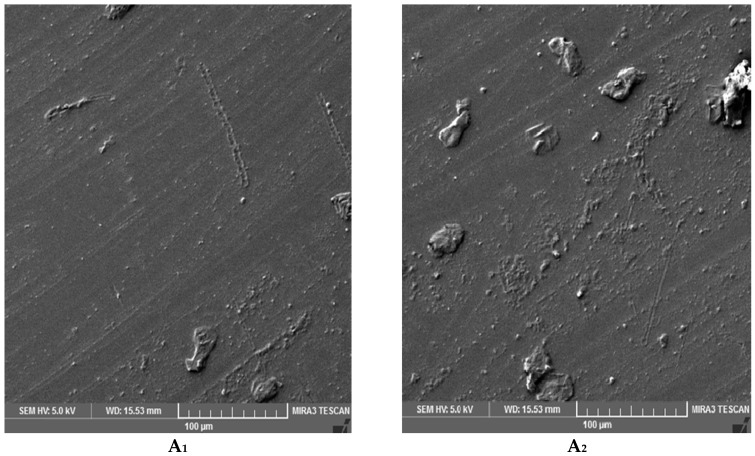

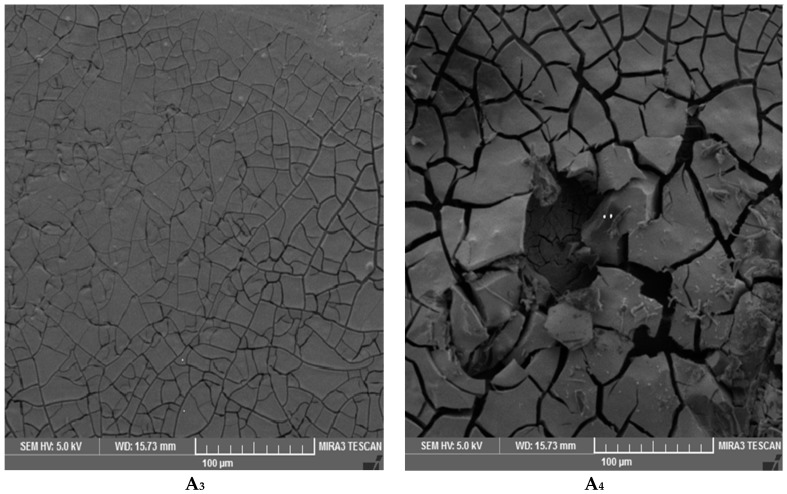
Scanning electron microscopy (SEM) images of sample A (DGEBA and Epikure F 205) after (**A_1_**) 0; (**A_2_**) 250; (**A_3_**) 500; and (**A_4_**) 800 h UV irradiation.

**Figure 12:**
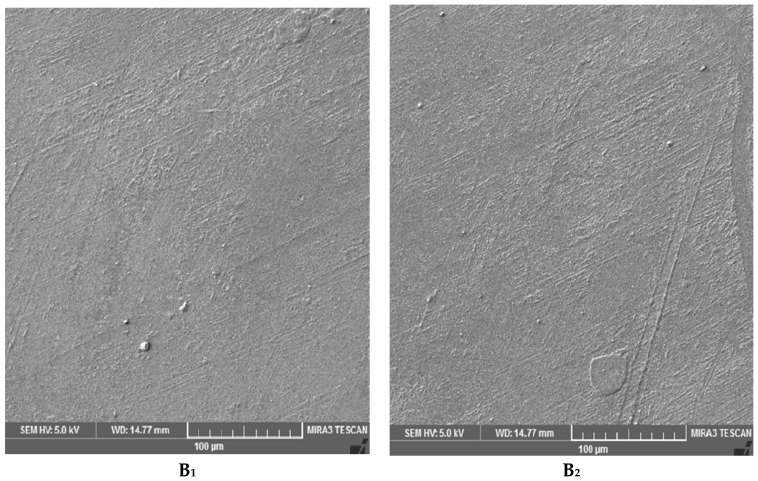

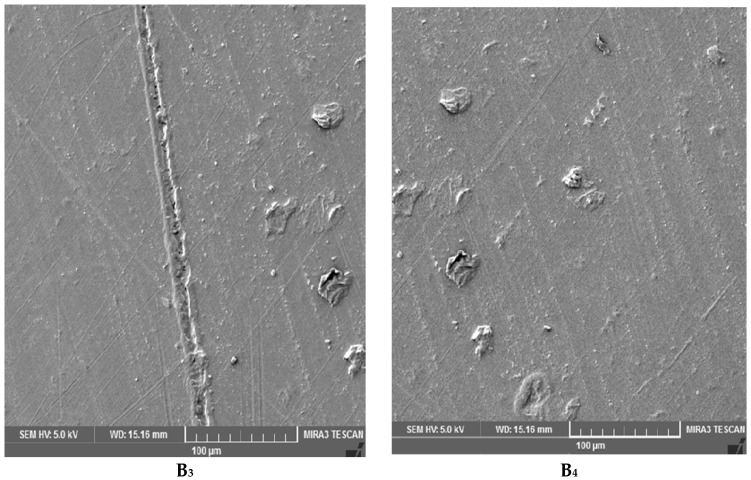
SEM images of sample B (DGEBA and Epikure F 205 with 3% Tinuvin 1130) after (**B_1_**) 0; (**B_2_**) 250; (**B_3_**) 500; and (**B_4_**) 800 h UV irradiation.

To study the effect of UV on the surface morphology of epoxy samples, SEM imaging was conducted. The surfaces of samples A (DGEBA and Epikure F 205) and B (DGEBA and Epikure F 205 with 3% Tinuvin 1130) after 0, 250, 500, 800 h of UV radiation are shown in Figure 11. As shown in Figure 11(A_1_), the surface of epoxy before UV radiation was fairly smooth, but after 250 h of UV radiation some nodes appeared on the surface attributed to the generation of volatile compounds, leading to the formation of ripples (Figure 11(A_2_)). After 500 h of UV exposure, the surface was covered with small cracks as seen in Figure 11(A_3_). As the exposure time increased to 800 h, surface cracks became wider and deeper, indicating severe damage (Figure 11(A_4_)). For samples containing Tinuvin 1130, no crack was observed after 250 and 500, and even 800 h of UV radiation, as can be seen in Figure 12. This difference in surface morphology after UV exposure was due to the presence of Tinuvin 1130. There were some visible nodes on the surface after 500 and 800 h of UV exposure. It was assumed that UV radiation resulted in relocation of organic UV absorbers from structure to the surface. Moreover, the SEM images further confirmed the mechanical properties discussed previously.

## 4. Conclusions

In summary, this study indicated that Tinuvin 1130, as an organic UV absorber, is an effective compound to decrease and even stop UV degradation in a cured epoxy-diamine system. FT-IR analyses showed that, after an 800-h UV radiation, an advanced UV degradation step occurred in the epoxy system without Tinuvin 1130, leading to formation of carboxylic acids. However, in the presence of Tinuvin 1130, UV damage could not progress well and it remained at the early stages. UV-Vis spectroscopy revealed that the epoxy system containing Tinuvin 1130 had a little yellowing after 800 h, whereas the pure epoxy system reached high levels of yellowing after the same duration. On comparison of tensile strength, pull-off strength, and Izod impact strength after UV radiation, it was found that Tinuvin 1130 effectively maintained the mechanical properties of the epoxy resin against UV light. SEM images also demonstrated that no cracks were seen on the surface of samples containing Tinuvin 1130 even after 800 h of UV exposure, while on the surface of samples without Tinuvin 1130, cracks were produced after 500 h of UV radiation and the surface was completely destroyed after 800 h of UV radiation. 

## Figures and Tables

**Table 1:** The areas under the visible range (400–700 nm) spectra of samples A (epoxy resin and Epikure F205) and B (epoxy resin and epikure F205 with 3% Tinuvin 1130) after 0, 250, 500, and 800 h UV radiation.

Sample	Area	Sample	Area
A_1_, 0 h	32.9	B_1_, 0 h	33.53
A_2_, 250 h	35.29	B_2_, 250 h	33.98
A_3_, 500 h	38.12	B_3_, 500 h	34.25
A_4_, 800 h	64.94	B_4_, 800 h	36.55

**Table 2:** The effect of UV light on tensile strength, tensile modulus, and elongation at break. Sample A (epoxy resin and Epikure F205) and Sample B (epoxy resin and Epikure F205 with 3% Tinuvin 1130).

Sample	Time Exposure to UV Light	Tensile Strength (MPa)	Tensile Modulus (MPa)	Elongation at Break (%)
A1	0 h	29.12 ± 2.4	1756 ± 11.5	1.75 ± 0.33
A2	250 h	27.87 ± 3.5	1750 ± 61.8	1.69 ± 0.15
A3	500 h	25.39 ± 1.4	1749 ± 45.4	1.42 ± 0.93
A4	800 h	20.25 ± 2.9	1747 ± 51.1	1.13 ± 0.67
B1	0 h	29.06 ± 6.9	1757 ± 74.7	1.74 ± 0.91
B2	250 h	28.38 ± 3.7	1755 ± 32.4	1.74 ± 0.42
B3	500 h	27.22 ± 2.5	1756 ± 63.9	1.63 ± 0.51
B4	800 h	25.59 ± 1.7	1751 ± 46.4	1.59 ± 0.37

**Table 3:** The results of pull-off strength samples A (DGEBA and Epikure F 205) and B (DGEBA and Epikure F 205 with 3% Tinuvin 1130) after 0, 250, 500, and 800 h UV irradiation.

Sample	Pull-Off Strength (MPa)
A1, 0 h	4.42 ± 0.4
A2, 250 h	4.33 ± 0.4
A3, 500 h	4.04 ± 0.1
A4, 800 h	3.52 ± 0.3
B1, 0 h	4.4 ± 0.3
B2, 250 h	4.35 ± 0.1
B3, 500 h	4.21 ± 0.3
B4, 800 h	4.12 ± 0.4
